# Neurofeedback Training Enables Voluntary Alteration of β-Band Power in the Subthalamic Nucleus of Individuals with Parkinson’s Disease

**DOI:** 10.1523/ENEURO.0144-19.2019

**Published:** 2019-04-23

**Authors:** Rosalind SE Carney

## Abstract

**Highlighted Research Paper:** Real-Time Neurofeedback to Modulate β-Band Power in the Subthalamic Nucleus in Parkinson’s Disease Patients, by Ryohei Fukuma, Takufumi Yanagisawa, Masataka Tanaka, Fumiaki Yoshida, Koichi Hosomi, Satoru Oshino, Naoki Tani and Haruhiko Kishima

Parkinson’s disease (PD) is a progressive neurodegenerative disorder caused by the death of dopamine-producing neurons in the substantia nigra. In the United States, almost 50,000 people receive a diagnosis of PD annually; PD affects more women than men, and the onset of symptoms typically occurs in the seventh decade of life (National Institute of Neurological Disorders and Stroke, 2017). Individuals with PD have difficulties with motor movements due to reduced dopaminergic innervation of the basal ganglia. Motor symptoms include tremor, slowness of movement, and uncoordinated movements. Treatment with l-DOPA, a synthetic precursor of dopamine, helps mitigate motor systems by decreasing β-band (13–30 Hz) oscillations in the subthalamic nucleus (STN), a structure that modulates basal ganglia output. In addition to treatment with l-DOPA, high-frequency stimulation [deep brain stimulation (DBS)] of the STN (STN-DBS) further improves motor symptoms of PD in humans and in animal models of PD (Hamani et al. 2004). The precise mechanisms of action of STN-DBS are not fully understood, but the clinical benefits occur almost immediately after the surgery to implant the electrodes and pacemaker (Hamani et al., 2017). Traditionally, STN-DBS for PD involved chronic neuronal stimulation, but currently an adaptive mode of stimulation is favored in which stimulation is modulated by concomitant feedback of pathological neural activity measures (Meidahl et al., 2017). In this manner, β-band oscillations can act as a feedback signal that determines how much stimulation is required as excessive stimulation can result in uncontrolled movements.

Adaptive STN-DBS shows that real-time neural activity acts as a neurofeedback signal that influences the plasticity of STN activity by modulating β-band power. Neurofeedback has previously been used to induce plastic changes in cortical activity (Ganguly et al., 2011; Wander et al., 2013; Orsborn et al., 2014; Emmert et al., 2016), including neuropathological states such as PD and stroke (Beuter et al., 2014; Buch et al., 2008; Ramos-Murguialday et al., 2013; Chaudhary et al., 2015). Therefore, Fukuma et al. (2018) speculated about whether the alteration of β-band power in the STN could be voluntarily induced by neurofeedback training in individuals with PD. Testing this experimental method required intracranial recording of real-time changes in β-band power and an output indicator that subjects could use to determine their success of voluntarily inducing β-band power changes. The authors devised an experiment in which the diameter of a black circle on a computer screen was scaled according to β-band power recorded by an electrode contact in the STN.

The subjects were eight individuals with PD (three male, five female) who were undergoing a routine surgery to replace pulse generators for bilateral STN-DBS implants. The subjects were administered local anesthesia and were instructed to remain as still as possible in the supine position. Any overt body movements were visually detected and noted by the researchers. Muscle movements of the hand were detected by electromyogram (EMG); the flexor digitorum superficialis and the extensor digitorum communis muscles of the hand were chosen as they are an antagonistic muscle pair that was easily accessible during the surgery.

The computer screen displaying the black circle was located 20–40 cm in front of each subject’s face (Fig. 1). For the 5 min prefeedback and postfeedback sessions, the subjects were instructed to close their eyes and to not fall asleep; these sessions measured the resting-state β-band power. For the feedback session, the subjects were asked to mentally focus their thoughts, in whatever manner possible, to make the black circle smaller in size. In each case, the highest β-band power signal from the DBS electrodes was selected to control circle size via computer software developed by the authors. The subjects could see the circle size in real time on the screen in front of them (Fig. 1). The subjects were unaware that they had been assigned to one of two groups: “down-training” and “up-training.” In the down-training condition, the diameter of the circle was scaled (0–1 range) proportionally to normalized (prefeedback resting state) β-band power, so that a voluntary reduction in β-band power made the circle smaller. Conversely, in the up-training condition, circle size was inversely scaled, such that an increase in β-band power made the circle smaller. Three prefeedback, feedback, and postfeedback sessions were conducted for each subject.

**Figure 1.**
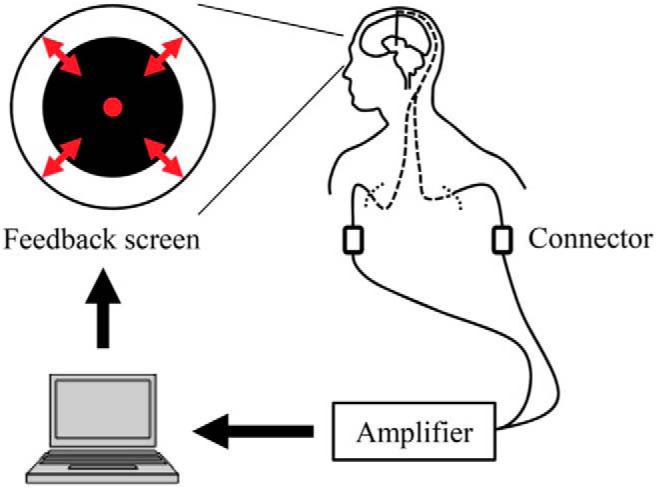
Feedback system overview. Signals from the DBS electrodes were acquired in real time. The diameter of the black circle on the computer screen was controlled based on the β-band power of the acquired bipolar signals from adjacent contacts that were selected in the prefeedback session (Fukuma et al., 2018, their Fig. 1).

In the down-training group, all four subjects successfully reduced β-band power to reduce the circle size. In the up-training group, two of four subjects successfully increased β-band power to reduce circle size. The authors suggest that increasing β-band power was more difficult to achieve given that β-band power is already high in individuals with PD. Overall, the results indicate that neurofeedback training enabled the subjects to voluntarily induce changes in β-band power in the STN (Fig. 2). The visual monitoring of the subjects confirmed that no overt hand movements were made. The EMG recordings revealed that voluntary modification of β-band power in the STN did not affect muscle activity, therefore, motor symptoms such as tremor were not alleviated during the neurofeedback session.

**Figure 2.**
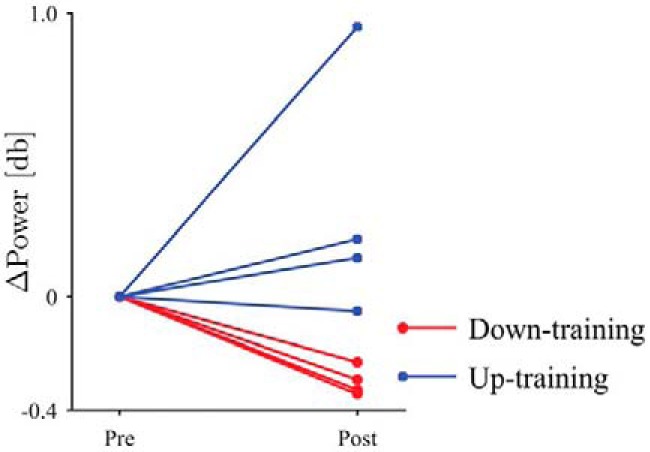
The difference in β-band power between the prefeedback and postfeedback sessions. The circular markers and red lines denote the down-training condition, whereas the square markers and blue lines indicate the up-training condition (Fukuma et al., 2018, their Fig. 4).

This is the first study that shows that individuals with PD can voluntarily alter β-band power in the STN. Future studies will use longer training sessions to determine whether neurofeedback can have an effect on motor activity or induce more lasting changes in β-band power. The authors hope that this experimental method could supplement treatment with l-DOPA and STN-DBS to mitigate further the adverse effects of abnormal β-band oscillations in individuals with PD. DBS is also used to treat epilepsy and obsessive-compulsive disorder and is currently being investigated as a potential treatment option for several other neurological disorders. Therefore, neurofeedback training could potentially become a combined neurosurgical and behavioral therapy intervention that could improve the lives of many people.
